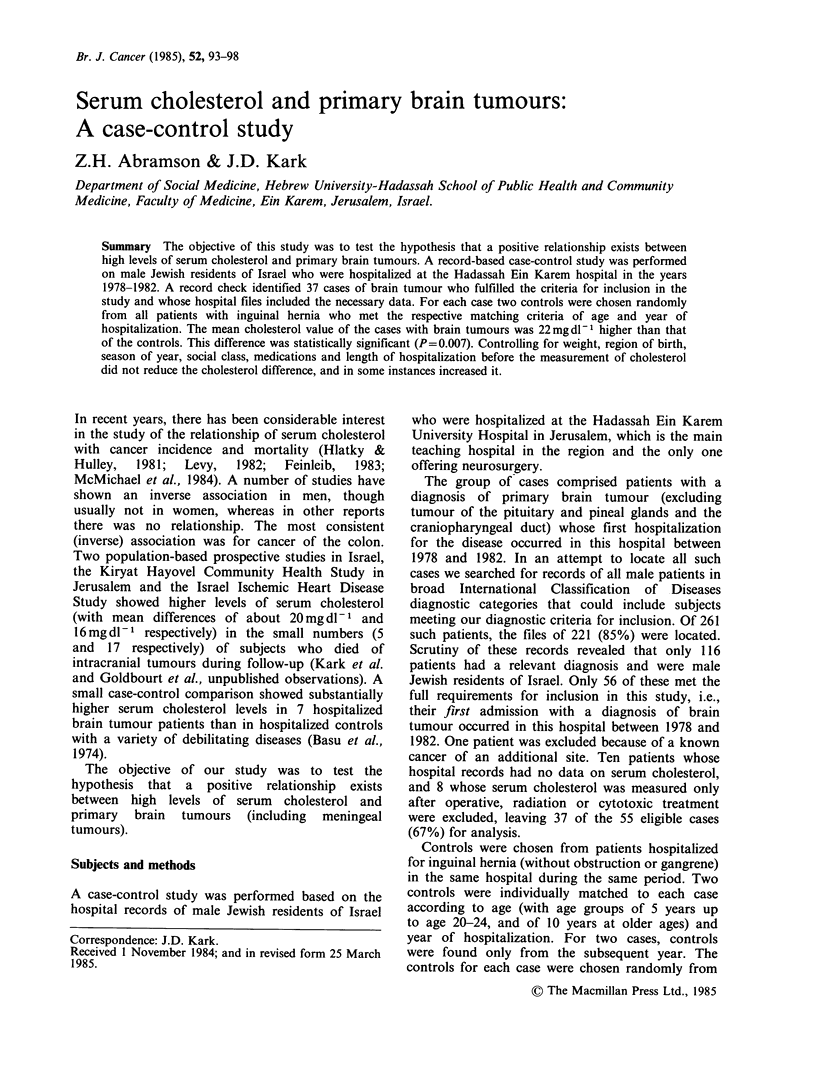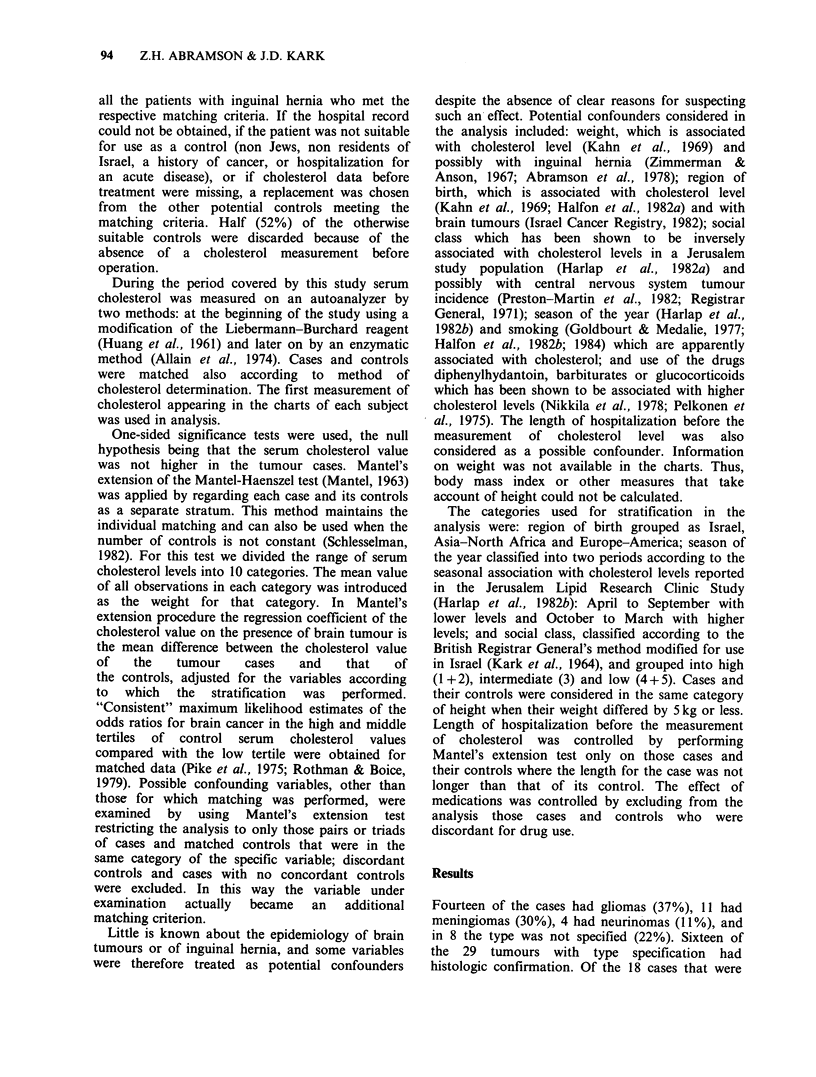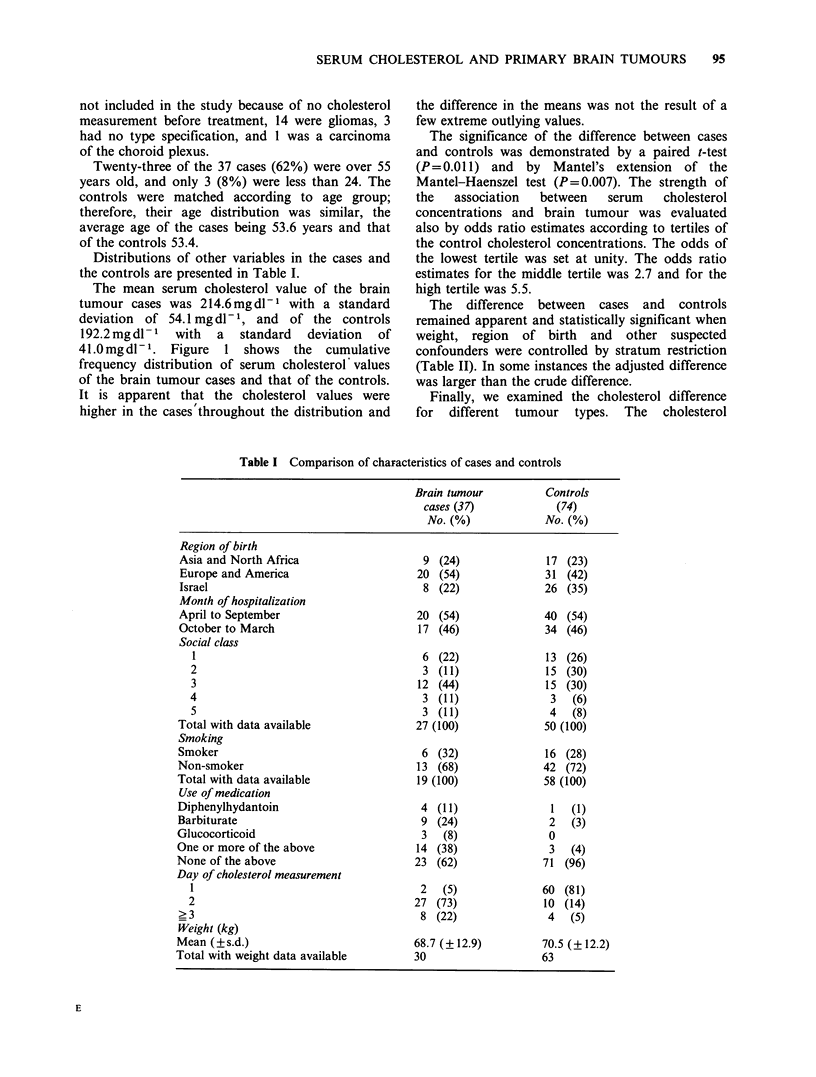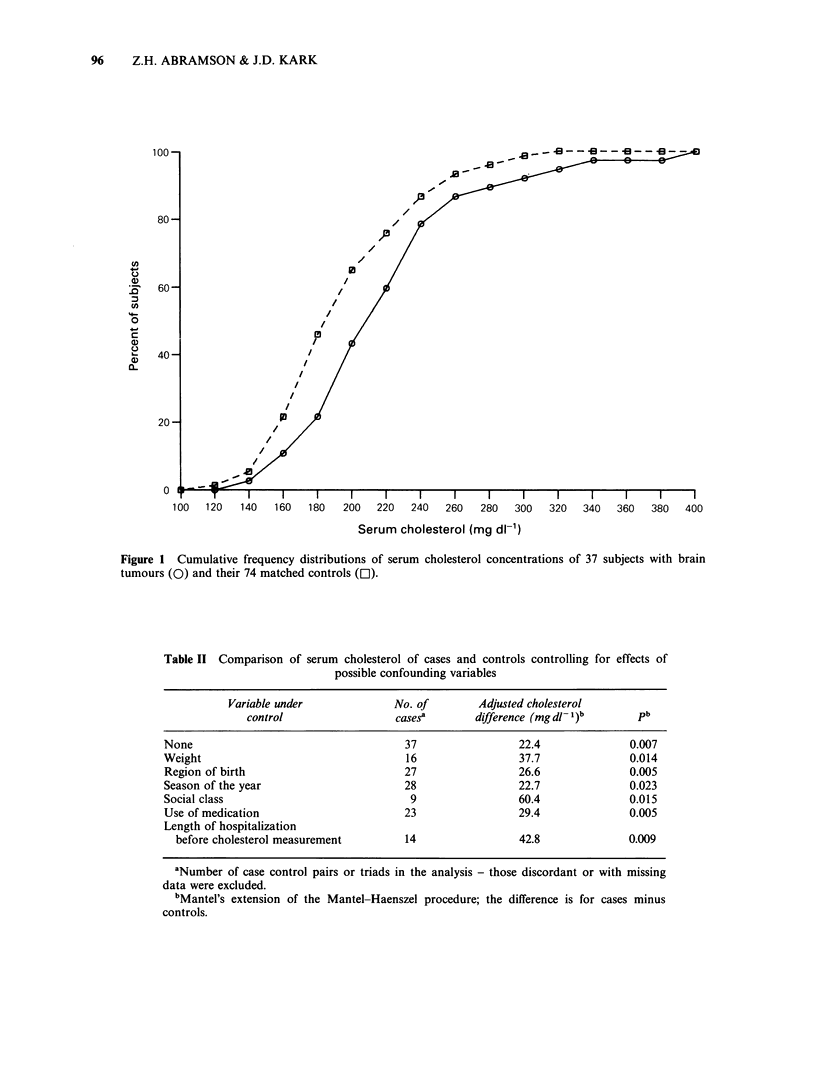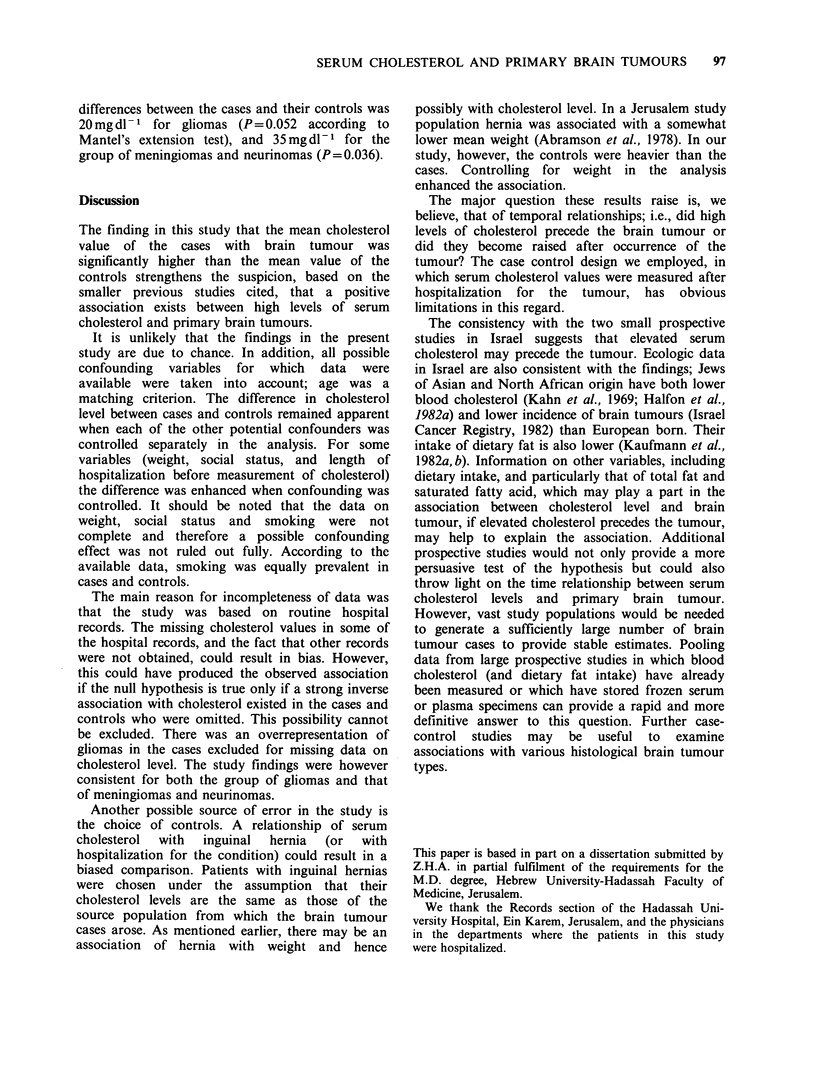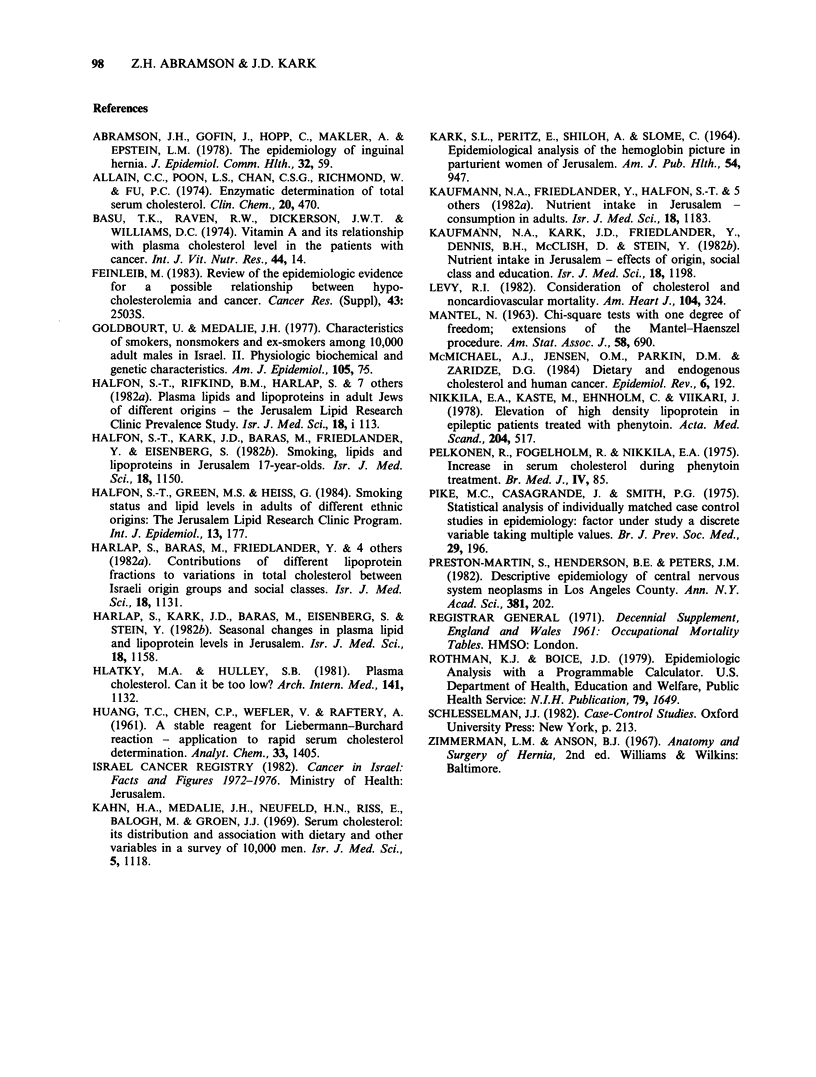# Serum cholesterol and primary brain tumours: a case-control study.

**DOI:** 10.1038/bjc.1985.154

**Published:** 1985-07

**Authors:** Z. H. Abramson, J. D. Kark

## Abstract

The objective of this study was to test the hypothesis that a positive relationship exists between high levels of serum cholesterol and primary brain tumours. A record-based case-control study was performed on male Jewish residents of Israel who were hospitalized at the Hadassah Ein Karem hospital in the years 1978-1982. A record check identified 37 cases of brain tumour who fulfilled the criteria for inclusion in the study and whose hospital files included the necessary data. For each case two controls were chosen randomly from all patients with inguinal hernia who met the respective matching criteria of age and year of hospitalization. The mean cholesterol value of the cases with brain tumours was 22 mg dl-1 higher than that of the controls. This difference was statistically significant (P = 0.007). Controlling for weight, region of birth, season of year, social class, medications and length of hospitalization before the measurement of cholesterol did not reduce the cholesterol difference, and in some instances increased it.


Br. J. Cancer (1985), **52**, 93–98

### Serum cholesterol and primary brain tumours: A case-control study

Z.H. Abramson & J.D. Kark

*Department of Social Medicine, Hebrew University-Hadassah School of Public Health and Community Medicine, Faculty of Medicine, Ein Karem, Jerusalem, Israel.*

**Summary** The objective of this study was to test the hypothesis that a positive relationship exists between high levels of serum cholesterol and primary brain tumours. A record-based case-control study was performed on male Jewish residents of Israel who were hospitalized at the Hadassah Ein Karem hospital in the years 1978–1982. A record check identified 37 cases of brain tumour who fulfilled the criteria for inclusion in the study and whose hospital files included the necessary data. For each case two controls were chosen randomly from all patients with inguinal hernia who met the respective matching criteria of age and year of hospitalization. The mean cholesterol value of the cases with brain tumours was 22 mg dl$^{-1}$ higher than that of the controls. This difference was statistically significant ($P = 0.007$). Controlling for weight, region of birth, season of year, social class, medications and length of hospitalization before the measurement of cholesterol did not reduce the cholesterol difference, and in some instances increased it.

In recent years, there has been considerable interest in the study of the relationship of serum cholesterol with cancer incidence and mortality (Hlatky & Hulley, 1981; Levy, 1982; Feinleib, 1983; McMichael *et al.*, 1984). A number of studies have shown an inverse association in men, though usually not in women, whereas in other reports there was no relationship. The most consistent (inverse) association was for cancer of the colon. Two population-based prospective studies in Israel, the Kiryat Hayovel Community Health Study in Jerusalem and the Israel Ischemic Heart Disease Study showed higher levels of serum cholesterol (with mean differences of about 20 mg dl$^{-1}$ and 16 mg dl$^{-1}$ respectively) in the small numbers (5 and 17 respectively) of subjects who died of intracranial tumours during follow-up (Kark *et al.* and Goldbourt *et al.*, unpublished observations). A small case-control comparison showed substantially higher serum cholesterol levels in 7 hospitalized brain tumour patients than in hospitalized controls with a variety of debilitating diseases (Basu *et al.*, 1974).

The objective of our study was to test the hypothesis that a positive relationship exists between high levels of serum cholesterol and primary brain tumours (including meningeal tumours).

### Subjects and methods

A case-control study was performed based on the hospital records of male Jewish residents of Israel who were hospitalized at the Hadassah Ein Karem University Hospital in Jerusalem, which is the main teaching hospital in the region and the only one offering neurosurgery.

The group of cases comprised patients with a diagnosis of primary brain tumour (excluding tumour of the pituitary and pineal glands and the craniopharyngeal duct) whose first hospitalization for the disease occurred in this hospital between 1978 and 1982. In an attempt to locate all such cases we searched for records of all male patients in broad International Classification of Diseases diagnostic categories that could include subjects meeting our diagnostic criteria for inclusion. Of 261 such patients, the files of 221 (85%) were located. Scrutiny of these records revealed that only 116 patients had a relevant diagnosis and were male Jewish residents of Israel. Only 56 of these met the full requirements for inclusion in this study, i.e., their *first* admission with a diagnosis of brain tumour occurred in this hospital between 1978 and 1982. One patient was excluded because of a known cancer of an additional site. Ten patients whose hospital records had no data on serum cholesterol, and 8 whose serum cholesterol was measured only after operative, radiation or cytotoxic treatment were excluded, leaving 37 of the 55 eligible cases (67%) for analysis.

Controls were chosen from patients hospitalized for inguinal hernia (without obstruction or gangrene) in the same hospital during the same period. Two controls were individually matched to each case according to age (with age groups of 5 years up to age 20–24, and of 10 years at older ages) and year of hospitalization. For two cases, controls were found only from the subsequent year. The controls for each case were chosen randomly from

Correspondence: J.D. Kark.
Received 1 November 1984; and in revised form 25 March 1985.

© The Macmillan Press Ltd., 1985

all the patients with inguinal hernia who met the respective matching criteria. If the hospital record could not be obtained, if the patient was not suitable for use as a control (non Jews, non residents of Israel, a history of cancer, or hospitalization for an acute disease), or if cholesterol data before treatment were missing, a replacement was chosen from the other potential controls meeting the matching criteria. Half (52%) of the otherwise suitable controls were discarded because of the absence of a cholesterol measurement before operation.

During the period covered by this study serum cholesterol was measured on an autoanalyzer by two methods: at the beginning of the study using a modification of the Liebermann–Burchard reagent (Huang *et al.*, 1961) and later on by an enzymatic method (Allain *et al.*, 1974). Cases and controls were matched also according to method of cholesterol determination. The first measurement of cholesterol appearing in the charts of each subject was used in analysis.

One-sided significance tests were used, the null hypothesis being that the serum cholesterol value was not higher in the tumour cases. Mantel's extension of the Mantel-Haenszel test (Mantel, 1963) was applied by regarding each case and its controls as a separate stratum. This method maintains the individual matching and can also be used when the number of controls is not constant (Schlesselman, 1982). For this test we divided the range of serum cholesterol levels into 10 categories. The mean value of all observations in each category was introduced as the weight for that category. In Mantel's extension procedure the regression coefficient of the cholesterol value on the presence of brain tumour is the mean difference between the cholesterol value of the tumour cases and that of the controls, adjusted for the variables according to which the stratification was performed. "Consistent" maximum likelihood estimates of the odds ratios for brain cancer in the high and middle tertiles of control serum cholesterol values compared with the low tertile were obtained for matched data (Pike *et al.*, 1975; Rothman & Boice, 1979). Possible confounding variables, other than those for which matching was performed, were examined by using Mantel's extension test restricting the analysis to only those pairs or triads of cases and matched controls that were in the same category of the specific variable; discordant controls and cases with no concordant controls were excluded. In this way the variable under examination actually became an additional matching criterion.

Little is known about the epidemiology of brain tumours or of inguinal hernia, and some variables were therefore treated as potential confounders despite the absence of clear reasons for suspecting such an effect. Potential confounders considered in the analysis included: weight, which is associated with cholesterol level (Kahn *et al.*, 1969) and possibly with inguinal hernia (Zimmerman & Anson, 1967; Abramson *et al.*, 1978); region of birth, which is associated with cholesterol level (Kahn *et al.*, 1969; Halfon *et al.*, 1982*a*) and with brain tumours (Israel Cancer Registry, 1982); social class which has been shown to be inversely associated with cholesterol levels in a Jerusalem study population (Harlap *et al.*, 1982*a*) and possibly with central nervous system tumour incidence (Preston–Martin *et al.*, 1982; Registrar General, 1971); season of the year (Harlap *et al.*, 1982*b*) and smoking (Goldbourt & Medalie, 1977; Halfon *et al.*, 1982*b*; 1984) which are apparently associated with cholesterol; and use of the drugs diphenylhydantoin, barbiturates or glucocorticoids which has been shown to be associated with higher cholesterol levels (Nikkila *et al.*, 1978; Pelkonen *et al.*, 1975). The length of hospitalization before the measurement of cholesterol level was also considered as a possible confounder. Information on weight was not available in the charts. Thus, body mass index or other measures that take account of height could not be calculated.

The categories used for stratification in the analysis were: region of birth grouped as Israel, Asia–North Africa and Europe–America; season of the year classified into two periods according to the seasonal association with cholesterol levels reported in the Jerusalem Lipid Research Clinic Study (Harlap *et al.*, 1982*b*): April to September with lower levels and October to March with higher levels; and social class, classified according to the British Registrar General's method modified for use in Israel (Kark *et al.*, 1964), and grouped into high (1+2), intermediate (3) and low (4+5). Cases and their controls were considered in the same category of height when their weight differed by 5 kg or less. Length of hospitalization before the measurement of cholesterol was controlled by performing Mantel's extension test only on those cases and their controls where the length for the case was not longer than that of its control. The effect of medications was controlled by excluding from the analysis those cases and controls who were discordant for drug use.

### Results

Fourteen of the cases had gliomas (37%), 11 had meningiomas (30%), 4 had neurinomas (11%), and in 8 the type was not specified (22%). Sixteen of the 29 tumours with type specification had histologic confirmation. Of the 18 cases that were not included in the study because of no cholesterol measurement before treatment, 14 were gliomas, 3 had no type specification, and 1 was a carcinoma of the choroid plexus.

Twenty-three of the 37 cases (62%) were over 55 years old, and only 3 (8%) were less than 24. The controls were matched according to age group; therefore, their age distribution was similar, the average age of the cases being 53.6 years and that of the controls 53.4.

Distributions of other variables in the cases and the controls are presented in Table I.

The mean serum cholesterol value of the brain tumour cases was 214.6 mg dl$^{-1}$ with a standard deviation of 54.1 mg dl$^{-1}$, and of the controls 192.2 mg dl$^{-1}$ with a standard deviation of 41.0 mg dl$^{-1}$. Figure 1 shows the cumulative frequency distribution of serum cholesterol values of the brain tumour cases and that of the controls. It is apparent that the cholesterol values were higher in the cases throughout the distribution and the difference in the means was not the result of a few extreme outlying values.

The significance of the difference between cases and controls was demonstrated by a paired *t*-test ($P=0.011$) and by Mantel's extension of the Mantel–Haenszel test ($P=0.007$). The strength of the association between serum cholesterol concentrations and brain tumour was evaluated also by odds ratio estimates according to tertiles of the control cholesterol concentrations. The odds of the lowest tertile was set at unity. The odds ratio estimates for the middle tertile was 2.7 and for the high tertile was 5.5.

The difference between cases and controls remained apparent and statistically significant when weight, region of birth and other suspected confounders were controlled by stratum restriction (Table II). In some instances the adjusted difference was larger than the crude difference.

Finally, we examined the cholesterol difference for different tumour types. The cholesterol

**Table I** Comparison of characteristics of cases and controls

| | *Brain tumour cases (37)* *No.* (%) | *Controls (74)* *No.* (%) |
|---|---|---|
| *Region of birth* | | |
| Asia and North Africa | 9 (24) | 17 (23) |
| Europe and America | 20 (54) | 31 (42) |
| Israel | 8 (22) | 26 (35) |
| *Month of hospitalization* | | |
| April to September | 20 (54) | 40 (54) |
| October to March | 17 (46) | 34 (46) |
| *Social class* | | |
| 1 | 6 (22) | 13 (26) |
| 2 | 3 (11) | 15 (30) |
| 3 | 12 (44) | 15 (30) |
| 4 | 3 (11) | 3 (6) |
| 5 | 3 (11) | 4 (8) |
| Total with data available | 27 (100) | 50 (100) |
| *Smoking* | | |
| Smoker | 6 (32) | 16 (28) |
| Non-smoker | 13 (68) | 42 (72) |
| Total with data available | 19 (100) | 58 (100) |
| *Use of medication* | | |
| Diphenylhydantoin | 4 (11) | 1 (1) |
| Barbiturate | 9 (24) | 2 (3) |
| Glucocorticoid | 3 (8) | 0 |
| One or more of the above | 14 (38) | 3 (4) |
| None of the above | 23 (62) | 71 (96) |
| *Day of cholesterol measurement* | | |
| 1 | 2 (5) | 60 (81) |
| 2 | 27 (73) | 10 (14) |
| ≧3 | 8 (22) | 4 (5) |
| *Weight (kg)* | | |
| Mean (±s.d.) | 68.7 (±12.9) | 70.5 (±12.2) |
| Total with weight data available | 30 | 63 |

**Figure 1** Cumulative frequency distributions of serum cholesterol concentrations of 37 subjects with brain tumours (○) and their 74 matched controls (□).

**Table II** Comparison of serum cholesterol of cases and controls controlling for effects of possible confounding variables

| *Variable under control* | *No. of cases*[a] | *Adjusted cholesterol difference (mg dl$^{-1}$)*[b] | $P^b$ |
|---|---|---|---|
| None | 37 | 22.4 | 0.007 |
| Weight | 16 | 37.7 | 0.014 |
| Region of birth | 27 | 26.6 | 0.005 |
| Season of the year | 28 | 22.7 | 0.023 |
| Social class | 9 | 60.4 | 0.015 |
| Use of medication | 23 | 29.4 | 0.005 |
| Length of hospitalization before cholesterol measurement | 14 | 42.8 | 0.009 |

[a]Number of case control pairs or triads in the analysis – those discordant or with missing data were excluded.

[b]Mantel's extension of the Mantel–Haenszel procedure; the difference is for cases minus controls.

differences between the cases and their controls was $20\,mg\,dl^{-1}$ for gliomas ($P=0.052$ according to Mantel's extension test), and $35\,mg\,dl^{-1}$ for the group of meningiomas and neurinomas ($P=0.036$).

### Discussion

The finding in this study that the mean cholesterol value of the cases with brain tumour was significantly higher than the mean value of the controls strengthens the suspicion, based on the smaller previous studies cited, that a positive association exists between high levels of serum cholesterol and primary brain tumours.

It is unlikely that the findings in the present study are due to chance. In addition, all possible confounding variables for which data were available were taken into account; age was a matching criterion. The difference in cholesterol level between cases and controls remained apparent when each of the other potential confounders was controlled separately in the analysis. For some variables (weight, social status, and length of hospitalization before measurement of cholesterol) the difference was enhanced when confounding was controlled. It should be noted that the data on weight, social status and smoking were not complete and therefore a possible confounding effect was not ruled out fully. According to the available data, smoking was equally prevalent in cases and controls.

The main reason for incompleteness of data was that the study was based on routine hospital records. The missing cholesterol values in some of the hospital records, and the fact that other records were not obtained, could result in bias. However, this could have produced the observed association if the null hypothesis is true only if a strong inverse association with cholesterol existed in the cases and controls who were omitted. This possibility cannot be excluded. There was an overrepresentation of gliomas in the cases excluded for missing data on cholesterol level. The study findings were however consistent for both the group of gliomas and that of meningiomas and neurinomas.

Another possible source of error in the study is the choice of controls. A relationship of serum cholesterol with inguinal hernia (or with hospitalization for the condition) could result in a biased comparison. Patients with inguinal hernias were chosen under the assumption that their cholesterol levels are the same as those of the source population from which the brain tumour cases arose. As mentioned earlier, there may be an association of hernia with weight and hence possibly with cholesterol level. In a Jerusalem study population hernia was associated with a somewhat lower mean weight (Abramson *et al.*, 1978). In our study, however, the controls were heavier than the cases. Controlling for weight in the analysis enhanced the association.

The major question these results raise is, we believe, that of temporal relationships; i.e., did high levels of cholesterol precede the brain tumour or did they become raised after occurrence of the tumour? The case control design we employed, in which serum cholesterol values were measured after hospitalization for the tumour, has obvious limitations in this regard.

The consistency with the two small prospective studies in Israel suggests that elevated serum cholesterol may precede the tumour. Ecologic data in Israel are also consistent with the findings; Jews of Asian and North African origin have both lower blood cholesterol (Kahn *et al.*, 1969; Halfon *et al.*, *1982a*) and lower incidence of brain tumours (Israel Cancer Registry, 1982) than European born. Their intake of dietary fat is also lower (Kaufmann *et al.*, 1982*a, b*). Information on other variables, including dietary intake, and particularly that of total fat and saturated fatty acid, which may play a part in the association between cholesterol level and brain tumour, if elevated cholesterol precedes the tumour, may help to explain the association. Additional prospective studies would not only provide a more persuasive test of the hypothesis but could also throw light on the time relationship between serum cholesterol levels and primary brain tumour. However, vast study populations would be needed to generate a sufficiently large number of brain tumour cases to provide stable estimates. Pooling data from large prospective studies in which blood cholesterol (and dietary fat intake) have already been measured or which have stored frozen serum or plasma specimens can provide a rapid and more definitive answer to this question. Further case-control studies may be useful to examine associations with various histological brain tumour types.

This paper is based in part on a dissertation submitted by Z.H.A. in partial fulfilment of the requirements for the M.D. degree, Hebrew University-Hadassah Faculty of Medicine, Jerusalem.

We thank the Records section of the Hadassah University Hospital, Ein Karem, Jerusalem, and the physicians in the departments where the patients in this study were hospitalized.

**References**

ABRAMSON, J.H., GOFIN, J., HOPP, C., MAKLER, A. & EPSTEIN, L.M. (1978). The epidemiology of inguinal hernia. *J. Epidemiol. Comm. Hlth.*, **32,** 59.

ALLAIN, C.C., POON, L.S., CHAN, C.S.G., RICHMOND, W. & FU, P.C. (1974). Enzymatic determination of total serum cholesterol. *Clin. Chem.*, **20,** 470.

BASU, T.K., RAVEN, R.W., DICKERSON, J.W.T. & WILLIAMS, D.C. (1974). Vitamin A and its relationship with plasma cholesterol level in the patients with cancer. *Int. J. Vit. Nutr. Res.*, **44,** 14.

FEINLEIB, M. (1983). Review of the epidemiologic evidence for a possible relationship between hypocholesterolemia and cancer. *Cancer Res.* (Suppl), **43:** 2503S.

GOLDBOURT, U. & MEDALIE, J.H. (1977). Characteristics of smokers, nonsmokers and ex-smokers among 10,000 adult males in Israel. II. Physiologic biochemical and genetic characteristics. *Am. J. Epidemiol.*, **105,** 75.

HALFON, S.-T., RIFKIND, B.M., HARLAP, S. & 7 others (1982*a*). Plasma lipids and lipoproteins in adult Jews of different origins – the Jerusalem Lipid Research Clinic Prevalence Study. *Isr. J. Med. Sci.*, **18,** i 113.

HALFON, S.-T., KARK, J.D., BARAS, M., FRIEDLANDER, Y. & EISENBERG, S. (1982*b*). Smoking, lipids and lipoproteins in Jerusalem 17-year-olds. *Isr. J. Med. Sci.*, **18,** 1150.

HALFON, S.-T., GREEN, M.S. & HEISS, G. (1984). Smoking status and lipid levels in adults of different ethnic origins: The Jerusalem Lipid Research Clinic Program. *Int. J. Epidemiol.*, **13,** 177.

HARLAP, S., BARAS, M., FRIEDLANDER, Y. & 4 others (1982*a*). Contributions of different lipoprotein fractions to variations in total cholesterol between Israeli origin groups and social classes. *Isr. J. Med. Sci.*, **18,** 1131.

HARLAP, S., KARK, J.D., BARAS, M., EISENBERG, S. & STEIN, Y. (1982*b*). Seasonal changes in plasma lipid and lipoprotein levels in Jerusalem. *Isr. J. Med. Sci.*, **18,** 1158.

HLATKY, M.A. & HULLEY, S.B. (1981). Plasma cholesterol. Can it be too low? *Arch. Intern. Med.*, **141,** 1132.

HUANG, T.C., CHEN, C.P., WEFLER, V. & RAFTERY, A. (1961). A stable reagent for Liebermann–Burchard reaction – application to rapid serum cholesterol determination. *Analyt. Chem.*, **33,** 1405.

ISRAEL CANCER REGISTRY (1982). *Cancer in Israel: Facts and Figures 1972–1976*. Ministry of Health: Jerusalem.

KAHN, H.A., MEDALIE, J.H., NEUFELD, H.N., RISS, E., BALOGH, M. & GROEN, J.J. (1969). Serum cholesterol: its distribution and association with dietary and other variables in a survey of 10,000 men. *Isr. J. Med. Sci.*, **5,** 1118.

KARK, S.L., PERITZ, E., SHILOH, A. & SLOME, C. (1964). Epidemiological analysis of the hemoglobin picture in parturient women of Jerusalem. *Am. J. Pub. Hlth.*, **54,** 947.

KAUFMANN, N.A., FRIEDLANDER, Y., HALFON, S.-T. & 5 others (1982*a*). Nutrient intake in Jerusalem – consumption in adults. *Isr. J. Med. Sci.*, **18,** 1183.

KAUFMANN, N.A., KARK, J.D., FRIEDLANDER, Y., DENNIS, B.H., McCLISH, D. & STEIN, Y. (1982*b*). Nutrient intake in Jerusalem – effects of origin, social class and education. *Isr. J. Med. Sci.*, **18,** 1198.

LEVY, R.I. (1982). Consideration of cholesterol and noncardiovascular mortality. *Am. Heart J.*, **104,** 324.

MANTEL, N. (1963). Chi-square tests with one degree of freedom; extensions of the Mantel–Haenszel procedure. *Am. Stat. Assoc. J.*, **58,** 690.

McMICHAEL, A.J., JENSEN, O.M., PARKIN, D.M. & ZARIDZE, D.G. (1984) Dietary and endogenous cholesterol and human cancer. *Epidemiol. Rev.*, **6,** 192.

NIKKILA, E.A., KASTE, M., EHNHOLM, C. & VIIKARI, J. (1978). Elevation of high density lipoprotein in epileptic patients treated with phenytoin. *Acta. Med. Scand.*, **204,** 517.

PELKONEN, R., FOGELHOLM, R. & NIKKILA, E.A. (1975). Increase in serum cholesterol during phenytoin treatment. *Br. Med. J.*, **IV,** 85.

PIKE, M.C., CASAGRANDE, J. & SMITH, P.G. (1975). Statistical analysis of individually matched case control studies in epidemiology: factor under study a discrete variable taking multiple values. *Br. J. Prev. Soc. Med.*, **29,** 196.

PRESTON-MARTIN, S., HENDERSON, B.E. & PETERS, J.M. (1982). Descriptive epidemiology of central nervous system neoplasms in Los Angeles County. *Ann. N.Y. Acad. Sci.*, **381,** 202.

REGISTRAR GENERAL (1971). *Decennial Supplement, England and Wales 1961: Occupational Mortality Tables*. HMSO: London.

ROTHMAN, K.J. & BOICE, J.D. (1979). Epidemiologic Analysis with a Programmable Calculator. U.S. Department of Health, Education and Welfare, Public Health Service: *N.I.H. Publication*, **79,** *1649*.

SCHLESSELMAN, J.J. (1982). *Case-Control Studies*. Oxford University Press: New York, p. 213.

ZIMMERMAN, L.M. & ANSON, B.J. (1967). *Anatomy and Surgery of Hernia*, 2nd ed. Williams & Wilkins: Baltimore.