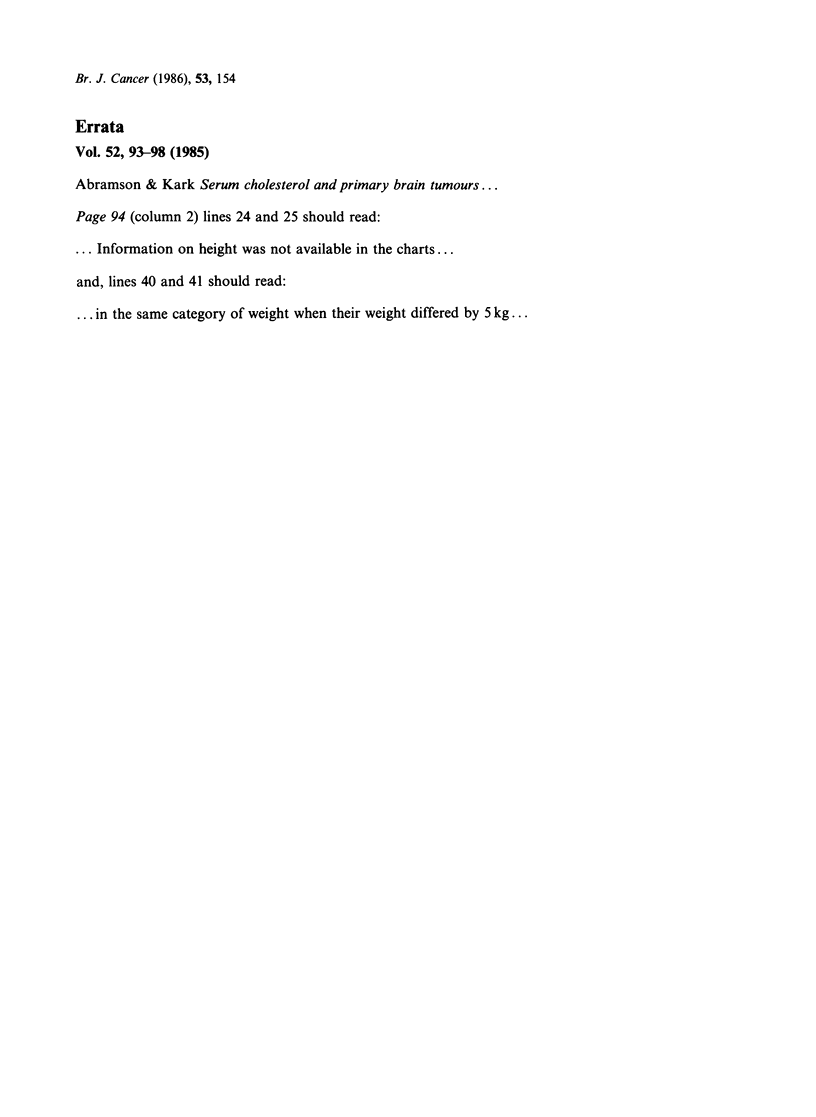# Errata

**Published:** 1986-01

**Authors:** 


*Br. J. Cancer* (1986), **53**, 154

### Errata

**Vol. 52, 93–98 (1985)**

Abramson & Kark *Serum cholesterol and primary brain tumours*...

*Page 94* (column 2) lines 24 and 25 should read:

... Information on height was not available in the charts...

and, lines 40 and 41 should read:

...in the same category of weight when their weight differed by 5 kg...